# Functional analysis finds differences on the muscle transcriptome of pigs fed an *n*-3 PUFA-enriched diet with or without antioxidant supplementations

**DOI:** 10.1371/journal.pone.0212449

**Published:** 2019-02-20

**Authors:** Marika Vitali, Rubina Sirri, Martina Zappaterra, Paolo Zambonelli, Giulia Giannini, Domenico Pietro Lo Fiego, Roberta Davoli

**Affiliations:** 1 Interdepartmental Centre for Industrial Agrifood Research (CIRI- AGRO), University of Bologna, Cesena, Italy; 2 Department of Agricultural and Food Sciences (DISTAL), University of Bologna, Bologna, Italy; 3 Department of Life Sciences, University of Modena and Reggio Emilia, Reggio Emilia, Italy; 4 Interdepartmental Research Centre for Agri-Food Biological Resources Improvement and Valorisation (BIOGEST-SITEIA), University of Modena and Reggio Emilia, Reggio Emilia, Italy; University of Illinois, UNITED STATES

## Abstract

Supplementing pig diets with *n*-3 polyunsaturated fatty acids (*n-*3 PUFA) may produce meat products with an increased *n*-3 fatty acid content, and the combined antioxidants addition could prevent lipid oxidation in the feed. However, to date, the effects of these bioactive compounds at the molecular level in porcine skeletal muscle are mostly unknown. This study aimed to analyse changes in the *Longissimus thoracis* transcriptome of 35 pigs fed three diets supplemented with: linseed (L); linseed, vitamin E and Selenium (LES) or linseed and plant-derived polyphenols (LPE). Pigs were reared from 80.8 ± 5.6 kg to 151.8 ± 9.9 kg. After slaughter, RNA-Seq was performed and 1182 differentially expressed genes (DEGs) were submitted to functional analysis. The L *vs* LES comparison did not show differences, while L *vs* LPE showed 1102 DEGs and LES *vs* LPE 80 DEGs. LPE compared to the other groups showed the highest number of up-regulated genes involved in preserving muscle metabolism and structure. Results enlighten that the combined supplementation of bioactive lipids (*n-*3 PUFA from linseed) with plant extracts as a source of polyphenols increases, compared to the only addition of linseed, the expression of genes involved in mRNA metabolic processes and transcriptional regulation, glucose uptake and, finally, in supporting muscle development and physiology. These results improve the knowledge of the biological effect of bioactive compounds in *Longissimus thoracis* muscle, and sustain the growing interest over their use in pig production.

## Introduction

Supplementing pig diets with *n-*3 PUFA is a strategy to obtain healthier meat products containing more unsaturated fatty acids (FAs) and a lower *n-*6/*n-*3 ratio, in view of the general concern on the high consumption of saturated FAs from red meat, which may increase the risk of disease such as type 2 diabetes and cardiovascular disease [1]. Several studies showed that supplementing farm animal diets with functional ingredients such as antioxidants can improve the nutritional quality of meat products by reducing lipid oxidation [2–7], and many functional additives containing vitamin E and polyphenols are already used in pig production. Despite that, knowledge of the effects of these nutrients at the molecular level is poorly known [8]. High-throughput technology such as next-generation sequencing of RNA (RNA-Seq) is presently an efficient suitable method in nutrigenomics studies in order to identify diet-induced changes in the transcriptome of a biological tissue [9]. In swine, few studies have investigated the effects of plant-derived bioactive compounds, such as *n-*3 PUFA and polyphenols or synthetic antioxidants on gene expression [10–14]. It is worth noting that both *n-*3 PUFA and antioxidants/polyphenols can have a positive role in human metabolism showing antioxidant and anti-inflammatory activity and a positive effect against obesity and insulin resistance [15–18]. However, the effects of antioxidants and polyphenols in the diet have been investigated mainly in rodents and only scarcely in farm animals so far [11,19]. In pigs, the few studies reported in literature evidenced that polyphenols may influence the expression of genes involved in lipid metabolism, inflammation and extracellular matrix remodelling [10,11] even if the role of these compounds in pig skeletal muscle needs to be better elucidated also at the molecular level [13]. The aim of the present research is to analyse the transcriptome of *Longissimus thoracis* muscle of pigs fed different diets supplemented with linseed, vitamin E and plant extracts as a source of polyphenols. This research aimed also to enlighten the effects of the different diets on skeletal muscle gene expression and to compare changes in the transcript profile among diets.

## Materials and methods

### Ethics approval

One of the partners of the research project was the Council for Agriculture and Agricultural Economy Analysis (Consiglio per la ricerca in agricoltura e l’analisi dell’economia agraria- CREA). This is a public body and member of the National Institutional Animal Care Committee. CREA stated with a decision included in the Report 2 of 2016 September, 14 that all the procedures performed in this study were in accomplishment with the Italian legislation, *D*.*Lgs 4 Marzo 2014 n*. *26 art*. *2 punto F*, and did not require further specific authorization. Moreover, all farming procedures followed the Council Directive 98/58/EC concerning the protection of animals kept for farming purposes, and Council Directive 2008/120/EC laying down minimum standards for the protection of pigs. Animal transport was performed according to Council Regulation (EC) No 1/2005 on the protection of animals during transport and related operations. Slaughter was performed at commercial abattoir following the Council Regulation (EC) n. 1099/2009 on the protection of animals at the time of killing and under the control of the Veterinary Service from the Italian Ministry of Health, as indicated in the Regulation (EU) 2017/625 of the European Parliament and of the Council on official controls and other official activities performed to ensure the application of food and feed law, rules on animal health and welfare, plant health and plant protection products. Authors declare that all the biological samples were collected from carcasses and none of them comes from live animals.

### Animals, diets and sampling

A total of 36 Italian Large White purebred pigs, 18 gilts and 18 barrows, were used for the study. The pigs were selected from a progeny of 258 piglets born from 21 sows and 3 boars registered to the herd book of the Italian National Association of Pig Breeders (ANAS, URL: http://www.anas.it/). Pigs were reared indoor in pens of three pigs each, on partially slatted floor. Each pen was provided with environmental enrichment devices constituted by metal chains with plastic discs inserted. The enrichments were manipulable and chewable but not edible, in order to avoid any influence over the effects of the diets. Water was always available with nipple drinkers installed inside each pen and their efficiency was tested twice *per* day. Animals were daily checked for any clinical sign of distress and the correct functioning of air ventilation, humidity and temperature was constantly monitored. No sanitary problems occurred during all the rearing period, with the exception of one pig dead in the finishing period due to an abdominal hernia. After weaning, the pigs were divided into 3 groups of 12 animals each, balanced for weight, father and sex. Pigs after weaning were all fed a standard diet until the starting of the trial at the average live weight of 80.8 ± 5.6 kg. During the trial each pig group was fed one of the following diets: a diet enriched with extruded linseed (source of *n-*3 PUFA) (L); a diet enriched with extruded linseed, vitamin E and selenium (LES); a diet enriched with extruded linseed and plant extracts from grape-skin and oregano (source of polyphenols) (LPE). Since one pig belonging to LPE group died before the end of the trial, this group was finally composed of 11 pigs. Diets were adjusted during the trial according to the average weight of pigs, as follows: during the first period (1st on Table 1), lasting from an average live weight of 80.8 ± 5.6 kg to 114.2 ± 10.7 kg, the amount of the supplied meal was calculated as 7.5% of the metabolic weight; during the finishing period (2nd on Table 1), ranging from 114.2 ± 10.7 kg to slaughter (at an average live weight of 151.8 ± 9.9 kg), the amount of the supplied meal was calculated as 8.5% of the pig metabolic weight. The composition of the diets administered and their nutritional contents are described in Table 1. The three diets were isoenergetic and isoproteic with the same lysine/digestible energy ratio. Diet formulation was done in order to reduce the content of linoleic acid and to increase linolenic acid, without damaging meat quality over lipid oxidation and microbiological susceptibility, as reported in the literature [20,21]. According to the same studies, the three diets were monocereal, based on barley as it has the lower content of linoleic acid, and extruded linseed was added to increase the content in *n*-3 PUFA. Linseed was extruded and the chemical composition resulted as follow: moisture (8%), crude fibre (25.0%), crude protein (20.2%), crude lipids (29.6%), and ashes (3.0%). The total content of α-Linolenic acid in linseed was 54.7%. The α-Linolenic acid content on the total *n*-3 PUFA (g per 100 g of total fatty acids) was 25.4% in all diets. Vitamin E addition in LES was done according to the study of Rossi et al. [22]. For what concerns plant extracts doses in LPE, since specific guidelines or reference standard do not exist in pig nutrition, plant-derived extracts were formulated according to the producers indication based on the human food industry. The analytical total content of polyphenols contained in plant extracts was 10.4 g/L for grape-skin extract and 3.9 g/L for oregano extract. Grape-skin extracts were produced by Enocianina Fornaciari s.n.c. (Reggio Emilia, Italy) and oregano extracts by Phenbiox s.r.l. (Bologna, Italy).

**Table 1:** Feed component and proximate composition (on a wet basis) of the three diets.

		L^(1)^		LES^(1)^		LPE^(1)^	
		1^st^^(2)^	2^nd^^(2)^	1^st^^(2)^	2^nd^^(2)^	1^st^^(2)^	2^nd^^(2)^
***Ingredients***							
**Extruded linseed**	%	5.00	5.00	5.00	5.00	5.00	5.00
**Barley meal**	%	80.50	86.60	80.30	86.40	80.50	86.60
**Soya bean meal**	%	11.00	5.00	11.00	5.00	11.00	5.00
**L-Lysine**	%	0.30	0.29	0.30	0.29	0.30	0.29
**DL-Methionine**	%	0.06	0.03	0.06	0.03	0.06	0.03
**L-Threonine**	%	0.05	0.03	0.05	0.03	0.05	0.03
**Calcium carbonate**	%	1.19	1.15	0.89	0.85	1.19	1.15
**Dicalcium phosphate**	%	1.00	1.00	1.00	1.00	1.00	1.00
**Salt (NaCl)**	%	0.40	0.40	0.40	0.40	0.40	0.40
**Vitamin/mineral pre-mix**	%	0.50	0.50	0.50	0.50	0.50	0.50
**Vitamin E and Selenium pre-mix**	%	0.00	0.00	0.50	0.50	0.00	0.00
**Plant extracts (Grape-skin + oregano)**	g *per* kg of feed	-	-	-	-	3.00+ 2.00	3.00+ 2.00
***Proximate composition***							
**Dry matter**	%	88.6	89.8	88.7	89.9	88.8	90.0
**Digestible energy**	kcal/kg DM	3255	3235	3248	3228	3255	3235
**Crude protein**	%	15.39	11.73	15.37	11.71	15.39	11.73
**Crude fat**	%	3.58	3.58	3.58	3.58	3.58	3.58
**Crude fibre**	%	4.62	4.48	4.61	4.47	4.62	4.48
**Ca**	%	0.82	0.79	0.82	0.79	0.82	0.79
**P**	%	0.55	0.53	0.55	0.53	0.55	0.53
***Fatty acids composition***	% of total FAs						
**C 14:0**	%	0.25	0.21	0.25	0.22	0.26	0.22
**C 16:0**	%	18.13	15.20	17.78	15.59	18.80	15.31
**C 16:1**	%	0.17	0.15	0.17	0.17	0.02	0.15
**C 18:0**	%	4.00	3.18	3.88	3.34	4.16	3.23
**C 18:1 *n-*9**	%	20.60	18.12	20.24	18.45	21.29	18.26
**C 18:2 *n-*6**	%	33.50	34.69	33.91	34.09	32.52	34.47
**C 18:3 *n-*3**	%	22.83	28.02	23.25	27.73	22.38	27.95
**C 20:1**	%	0.53	0.41	0.52	0.42	0.57	0.41

^(1)^L = standard diet supplemented with extruded linseed (source of *n-*3 PUFA); LES = standard diet supplemented with extruded linseed, vitamin E and selenium; LPE = standard diet supplemented with extruded linseed and plant extracts (source of polyphenols).

^(2)^1st = feed administered from an average weight of 80 kg to 115kg; 2nd = feed administered from an average weight of 115 kg to slaughter.

At the end of the trial, animals were transported to a commercial abattoir and slaughtered in two batches, at an interval of 14 days. Each batch was composed by half of the pigs from each group (*i*.*e*. six pigs per group were sacrificed for each slaughter day). Pigs were weighted the day before the slaughtering and the six heaviest pigs in each group were chosen to be sacrificed in the first slaughter batch. Slaughter was performed at the abattoir O.P.A.S. Società Cooperativa Agricola, Via Guastalla 21A, 41012 Carpi MO (Italy). Pigs were electrically stunned and were sacrificed by jugulation on prone position. All slaughter procedures were monitored by the veterinary team appointed by the Italian Ministry of Health. At the end of the slaughter line, a sample of about 50 g of *Longissimus thoracis* muscle was taken from each pig carcass and immediately frozen in liquid nitrogen. Samples were then stored at -80°C pending RNA extraction. *Longissimus thoracis* muscle was chosen as it is one of the main cuts intended for fresh meat consumption and it is one of the most studied in pork production.

### RNA extraction, library preparation and sequencing

Total RNA was extracted using a standard RNA protocol with TRIzol (Invitrogen, Carlsbad, CA, USA) from 30 mg of *Longissimus thoracis* muscle. The RNA concentration was then evaluated in each sample with an ND-1000 spectrophotometer (NanoDrop Technologies) and the quality was assessed with the Agilent 2100 Bioanalyzer through Agilent RNA 6000 nano kit (Agilent Technologies, Santa Clara, CA, USA). Samples were processed only if the RIN quality was > 7. Next-generation sequencing analysis was performed by the external service Genomix4life S.R.L. (Baronissi, Salerno, Italy). The indexed libraries were prepared from 1 μg of purified RNA from each sample with TruSeq Stranded mRNA (Illumina, San Diego, CA, USA) Library Prep Kit. The libraries were quantified using the Agilent 2100 Bioanalyzer (Agilent Technologies, Santa Clara, CA, USA) and divided into 3 pools such that each index-tagged sample was present in equimolar amounts, with a final concentration of the pooled samples of 2nM. The pooled samples were submitted to cluster generation and sequencing using an Illumina HiSeq 2500 System (Illumina, San Diego, CA, USA) in a 2x100 paired-end (RNA-Seq) format loading the pool on a single lane. The raw sequence files generated are in FASTQ format. The sequences obtained by RNA-Seq analysis from the present study can be retrieved from Annotare database under the accession number E-MTAB-7131.

### RNA-Seq data processing

RNA-Seq data processing and gene expression analysis were performed by the external service MENTOTHEC s.r.l. (Naples, Italy). Quality control of the raw reads was performed through the FastQC tool (http://www.bioinformatics.babraham.ac.uk/projects/fastqc/), which generates a report for each sample read set. All reads were trimmed using the BBDuk software (https://jgi.doe.gov/data-and-tools/bbtools/) to eliminate Illumina adapters and bases with a quality Phred score lower than 25; only the reads with a length higher than 35 nucleotides were kept after trimming. The high-quality reads were aligned to the swine genome (Sscrofa11.1) using STAR aligner (version 2.5.2b) [23].

For gene expression analysis, the program featureCounts implemented in Subread software (version 1.5.1 [24]) was used to calculate the gene expression values as raw fragment counts, followed by FPKM (Fragments Per Kilobase of transcript per Million mapped reads) calculation with EdgeR [25]. Then the genes were assessed for differential expression (DE) among pairs of groups: L *vs*. LPE, LES *vs*. LPE, L *vs*. LES for a total of three comparisons. These comparisons were performed with NOISeq R/Bioc package [26], applying a TMM (Trimmed Mean of M values) normalization, removing the genes with less than 1 CPM (Counts Per Million) in all the samples and after applying the ARSyN (ASCA Removal of Systematic Noise for sequencing data) correction method using the dietary groups as factors. The posterior probabilities of differential expression were converted to false discovery rate (FDR) as showed in the NOISeq manual. Differentially expressed genes were considered statistically significant with the FDR *P-*value ≤ 0.05.

### Validation using RT-qPCR

Validation was performed using quantitative Real-Time PCR (RT-qPCR) standard curve method [27]. Five genes were selected among the differentially expressed genes (DEGs) and used to validate RNA-Seq data. The synthesis of cDNA was performed from 1μg of RNA using the ImProm-II Reverse Transcription System (Promega Corporation, Milan, Italy), resulting in 20 μl of cDNA solution. RT-qPCR was performed on Rotor Gene 6000 (Corbett Life Science, Concorde, New South Wales, Australia) using 5 μl of SYBR Premix Ex Taq (TAKARA Bio INC, Olsu, Shiga, Japan), 10 pmol of each primer, 2 μl of cDNA template diluted 1:10 in nuclease-free water. RT-qPCR was performed using a two-step amplification constituted by a denaturation phase of 95°C for 5 seconds, followed by an annealing-extension phase at temperatures optimized per each primer couple for 20 seconds (annealing temperatures for each primer couple were reported in S1 Table). Each cycle was repeated for 40 times. The variation coefficient (CV = standard deviation of the crossing points/average of the crossing points) of the replicated analysis for each sample (three in 2 different cycles of RT-qPCR) was set at 0.2 as maximum level accepted. *Beta-2-microglobulin* (*B2M*) and *hypoxanthine phosphoribosyltransferase 1* (*HPRT1*), were used as reference genes. The expression levels of the five selected genes were then calculated using the standard curve methods, as described in Zappaterra *et al*. [28]. Standard curves were obtained amplifying 12 progressive dilutions (from 10^9^ to 25 molecules/μl) of a known concentration of a cDNA sample, obtained by PCR. The absence of unspecific amplicons during RT-qPCR on Rotor-Gene 6000 was tested using the melting step after the cycling. Pearson’s correlations were then calculated between RT-qPCR and RNA-Seq expression data for the five tested genes using the R software [29]. Correlation coefficient (R) was considered significant if *P* ≤ 0.05.

### Functional analysis of differentially expressed genes

For the analysis, only DEGs presenting a Log_2_ fold change (Log_2_FC) ≥ 0.30 or ≤ -0.30 and an FDR adjusted *P* ≤ 0.05 were considered. To compensate poor pig gene annotation, the *Homo sapiens* background was applied, so the gene IDs were converted to human gene IDs using BioMart–Ensembl (URL: https://www.ensembl.org/biomart) prior to proceeding with the functional analysis. The functional analyses were performed separately on each pairwise comparison L *vs* LPE, L *vs* LES, LES *vs* LPE.

The functional enrichment analysis was carried out using two software: Cytoscape v3.5.1 software (Institute for Genomics and Bioinformatics, Graz University of Technology, Graz, Austria) and DAVID Functional Annotation Tool v. 6.8 (URL: https://david.ncifcrf.gov/).

Cytoscape analysis started building a network of DEGs using the GeneMANIA plug-in [30] and then functional analysis was performed using the ClueGO plug-in [31]. For each diet comparison, the ClueGO plug-in divided the significant DEGs into different functional groups having different *P*-values. Each group contained the pathways and biological processes (BPs) regrouped in functional groups according to terms similarities. Each pathway and BPs in each functional group had different *P*-values and could contain both up and down-regulated DEGs. If the percentage of up-regulated DEGs were the majority, that pathway/biological process was assigned to *cluster #1*, thus considered up-regulated; if the down-regulated DEGs were the majority, that pathway/biological process was assigned to *cluster #2*, thus considered down-regulated; finally if the percentage of both up-regulated and down-regulated DEGs in a pathway/biological process was ranging between 40–60%, the cluster was called *none specific cluster*. The statistical method was set at right-sided hypergeometric distribution, and Bonferroni’s *P*-value correction was used. Minimum clustering was set at *P* ≤ 0.05 and minimum k-score at 0.4. The BPs ontology and KEGG and REACTOME pathways were used as databases for the functional analysis. Gene ontology (GO) levels were set from 6 to 8, and a minimum number of genes per cluster was set at 5 (in case the number of DEGs in a cluster was minor than 5, the maximum number of available genes was set in that cluster). To graphically represent the data obtained, REVIGO online tool (URL: http://revigo.irb.hr/) was employed to summarize the enriched GO terms and, when necessary, also the pathways were summarized by selecting the higher level in REACTOME pathway hierarchy (URL: https://reactome.org/user/guide/pathway-browser). Subsequently, CytoHubba and CluePedia plug-ins were applied to select and display in the figures the hub DEGs with the aim to visualize the interaction between the most significant DEGs and their related pathways and BPs. Only the pathways and BPs linked to these selected hub genes were chosen to visualize the interaction between DEGs and GO Terms in the presented figures.

The DAVID Functional Annotation Tool v. 6.8 (URL: https://david.ncifcrf.gov/) was directed to identify Pathways from the Kyoto Encyclopedia of Genes and Genomes (KEGG) and REACTOME database and the Gene Ontology GOTERM Biological Process [32]. The genes were uploaded using the Human genome as background. *P*-value of the term enrichment was evaluated using Benjamini’s correction and *P* ≤ 0.05 was considered significant. The up- and down-regulated DEGs were analysed separately in each diet comparison.

## Results

### Differentially expressed genes

**Fig 1:**
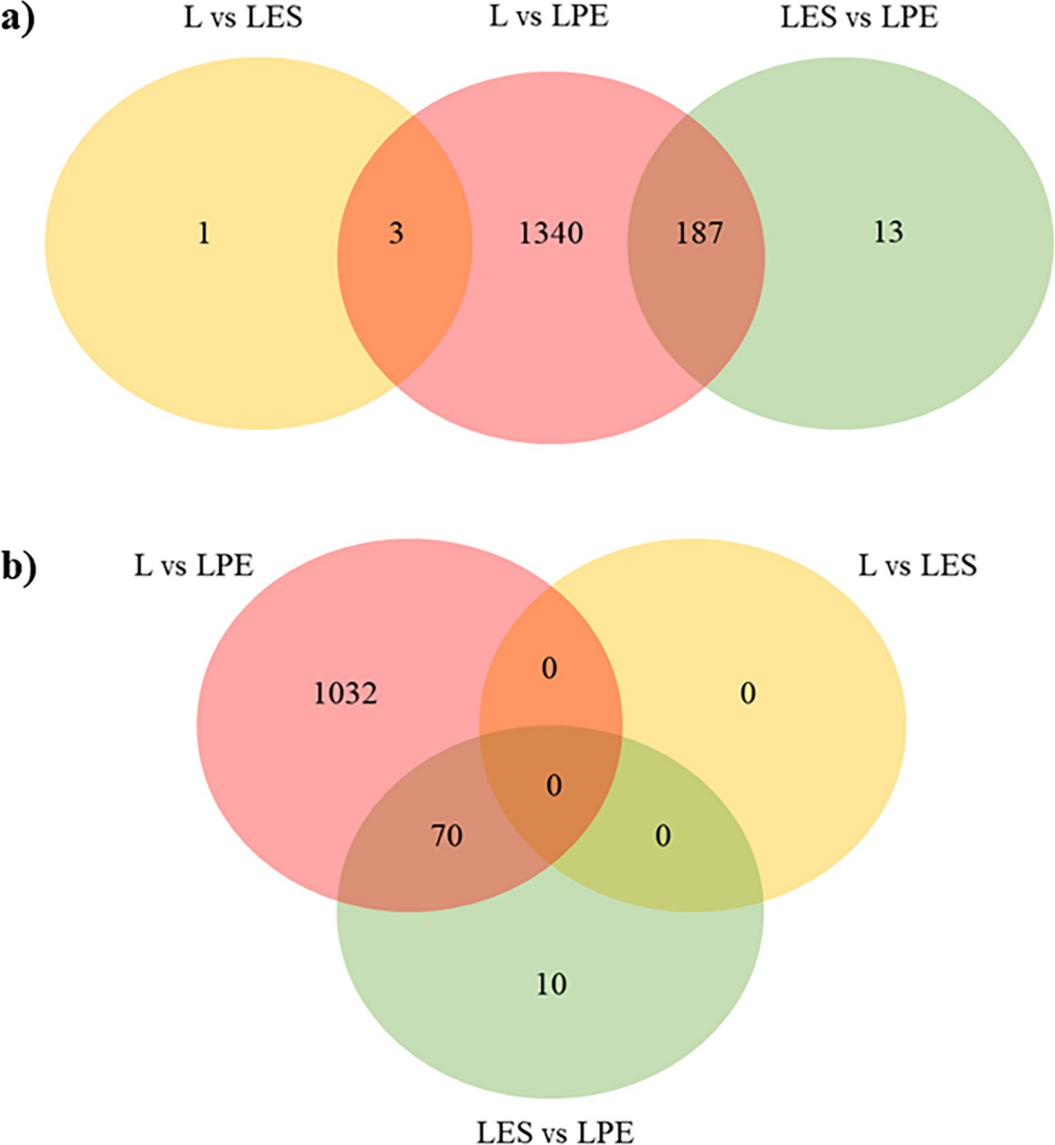
**Venn diagram showing the distribution of DEGs in the three diet comparisons before (a) and after (b) the Log_2_FC cut-off application**.

The obtained total reads are reported in S2 Table. RNA-Seq analysis resulted in a total of 1544 DEGs among the three diet comparisons (L *vs* LES, L *vs* LPE, LES *vs* LPE) with several DEGs common in more than one comparison, for a total of 1734 DEGs (Fig 1; Table 2; S3 Table). The comparison L *vs* LPE showed to have the highest number of DEGs, while the L *vs* LES showed the lowest number of DEGs with only four differentially expressed genes. Before submitting genes to functional analysis, a Log_2_FC cut-off ≥ +0.30 or ≤ -0.30 was applied. After the cut-off application, the number of genes was reduced to 1112 differentially expressed genes (DEGs) and then submitted to functional analysis (Fig 1, Table 2). The L *vs* LES comparison did not present DEGs, therefore it was not considered for the functional analysis. The total number of genes in Table 2 includes also the DEGs common to more than one comparison. The complete list of significant DEGs and the information about the Log_2_FC and FDR adjusted *P*-values are reported in S3 Table.

**Table 2:** Up- and down-regulated DEGs in group comparisons before and after setting the Log_2_FC cut-off. The total number of DEGs (1182) considers also genes common to more than one comparison.

	L-LES		L-LPE		LES-LPE	
	All	Cut-off	All	Cut-off	All	Cut-off
**Up-regulated**	4	0	726	628	21	21
**Down-regulated**	0	0	804	474	179	59
**Total**	4	0	1530	1102	200	80

In LES *vs* LPE, 70 out of 80 DEGs are the same genes found in L *vs* LPE comparison (Fig 1), also showing the same expression trend (*e*.*g*. the genes up-regulated in LPE compared to L were also up-regulated in LPE with respect to LES, and *vice versa*) (S3 Table). Only ten genes *(DMPK*, *DNAJA4*, *HSPA8*, *HSP90AA1*, *HSP90AB1*, *IER5*, *PDE4B*, *PSMC1*, *SLC20A1*, *STIP1)* were not common between the two comparisons, and they are all up-regulated in LPE compared to LES.

### Functional analysis with Cytoscape

**Fig 2:**
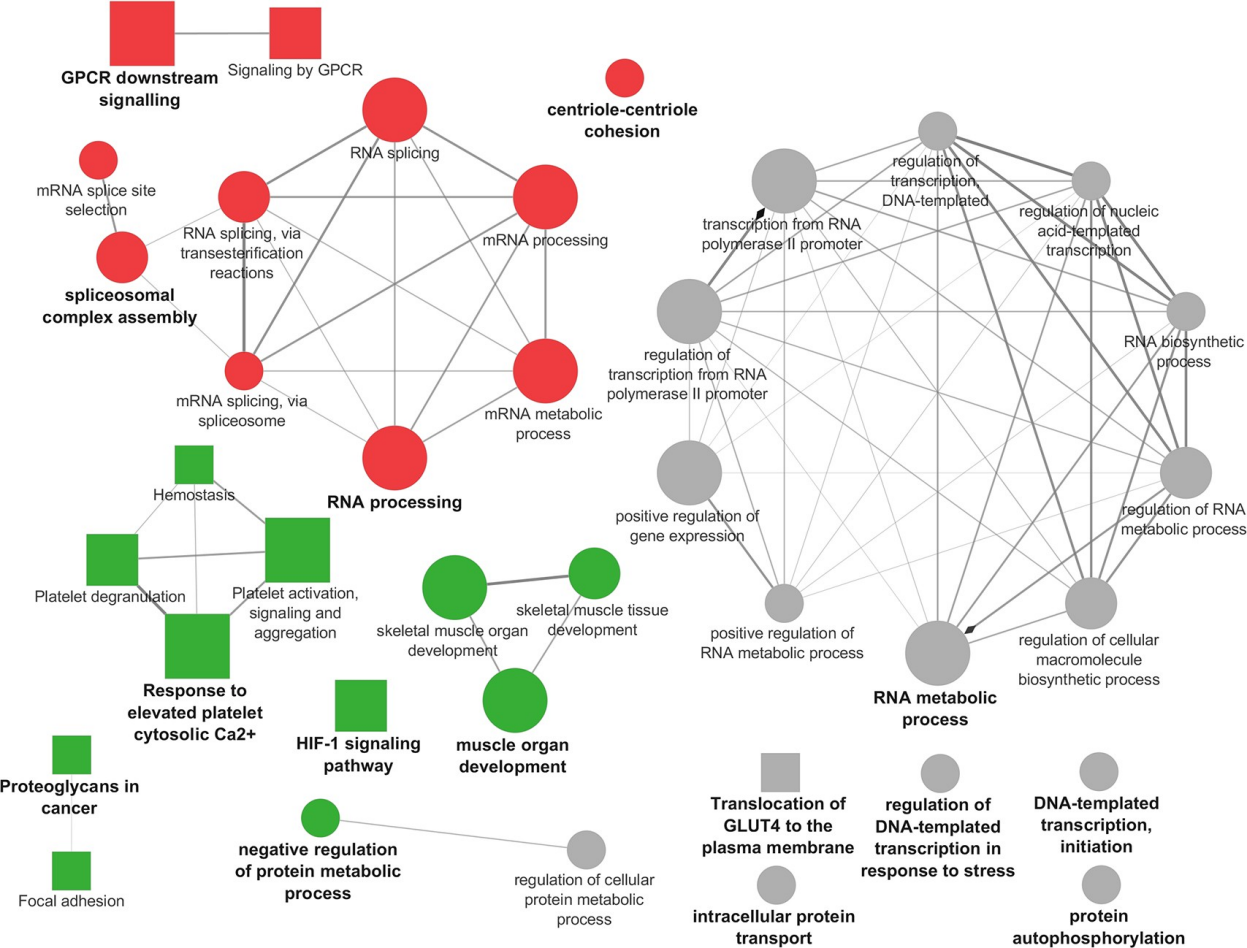
Cytoscape functional analysis of L *vs* LPE comparison. squares = pathways; circles = biological processes; shape size = according to the *P*-value of the term in its own group; red colour = up-regulated (*cluster #1*); green colour = down-regulated (*cluster #2*); grey colour = same number of up- and down-regulated genes (*none specific cluster*); font size = according to the *P*-value of the term in its own group; interaction line thickness = according to Kappa Score value, represents the strength of the interactions, lighter colour corresponds to a lower strength while darker colour to a higher strength.

**Fig 3:**
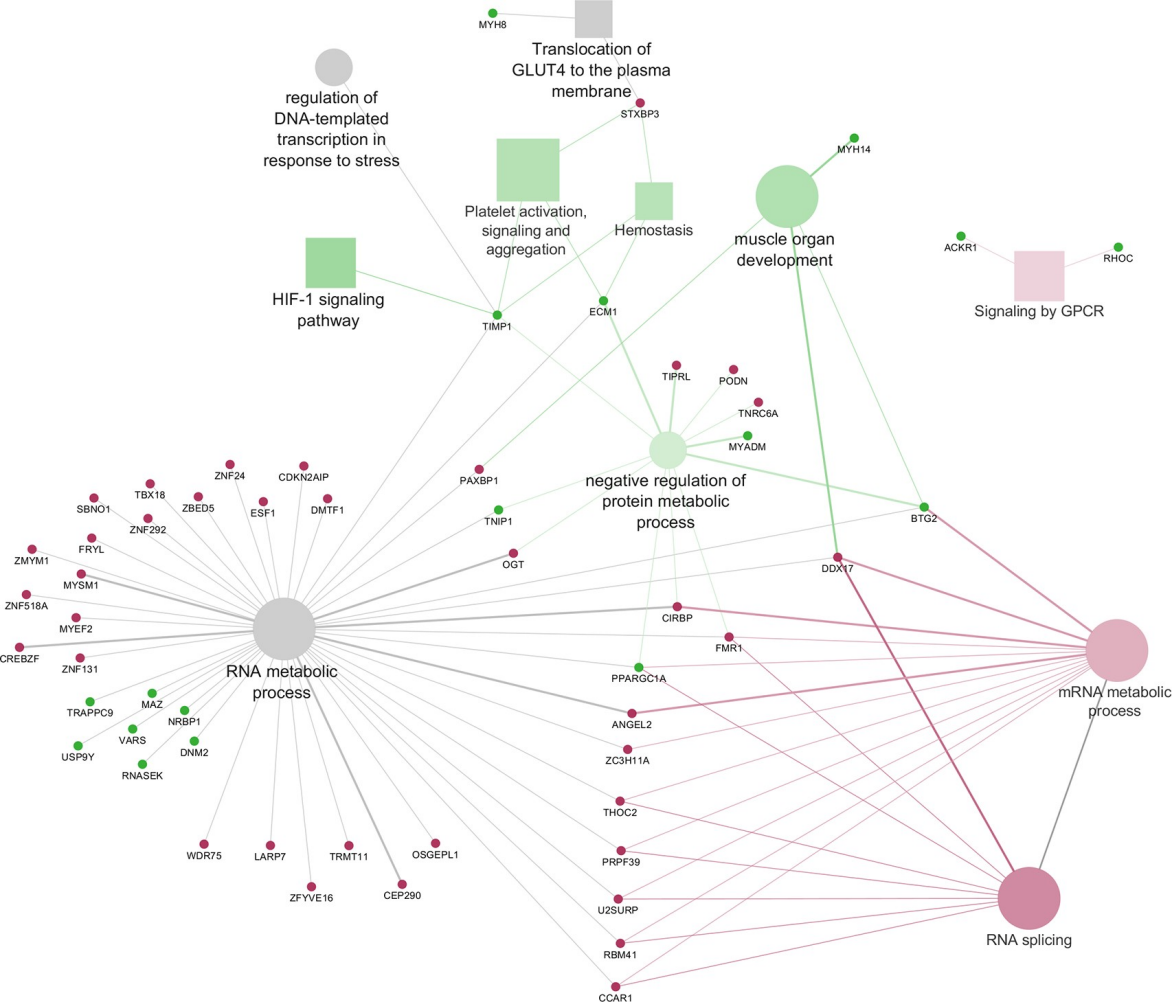
Cytoscape functional analysis of L *vs* LPE comparison displaying hub DEGs. Significant GO terms are graphically summarized using REVIGO. squares = pathways; circles = biological processes; shape size = according to the *P*-value of the term in its own group; red colour = up-regulated (*cluster #1*); green colour = down-regulated (*cluster #2*); grey colour = same number of up- and down-regulated genes (*None specific cluster*); fill colour transparency = according to the percentage of genes belonging to the term, lighter colour corresponds to a lower percentage while darker colour to a higher percentage; font size = according to the *P*-value of the term in its own group; interaction line thickness = according to Kappa Score value, represents the strength of the interactions, lighter colour corresponds to a lower strength while darker colour to a higher strength.

Results from the functional analysis showed that in L *vs* LPE, the enriched pathways and biological processes included in the *cluster #1* (majority of DEGs up-regulated by L) were represented by four functional groups (Figs 2 and 3, S4 Table). For each of these groups the most significant pathway/biological process (leading term) was identified, according to *P*-value, namely: “RNA processing” (*P* = 0.00001), “GPCR downstream signaling” (*P* = 0.00002), “RNA splicing via transesterification reactions” (*P* = 0.004), “Centriole-centriole cohesion” (*P* = 0.03). The enriched pathways and biological processes included in *cluster #2* (the majority of DEGs down-regulated by L, thus up-regulated in LPE) were represented by five functional groups (Figs 2 and 3, S4 Table). For each of these five groups, the most significant pathway/biological process (leading term) was identified, namely: “Muscle organ development” (*P* = 0.00005), “Response to elevated platelet cytosolic Ca^2+^” (*P* = 0.0003), “HIF-1 signaling pathway” (*P* = 0.002), “Proteoglycans in cancer” (*P* = 0.01), “Negative regulation of protein metabolic process” (*P* = 0.02). Finally, the enriched pathways and biological processes included in the *none specific cluster* were characterized by seven functional groups (Figs 2 and 3, S4 Table). For each of these seven groups it was identified the leading term according to *P*-value: “RNA metabolic process” (*P* = 1.79E-06), “Translocation of GLUT4 to the plasma membrane” (*P* = 0.01), “Intracellular protein transport” (*P* = 0.01), “Regulation of DNA-templated transcription in response to stress” (*P* = 0.01), “Regulation of cellular protein metabolic process” (*P* = 0.02), “DNA-templated transcription, initiation” (*P* = 0.04), “Protein autophosphorylation” (*P* = 0.04).

**Fig 4:**
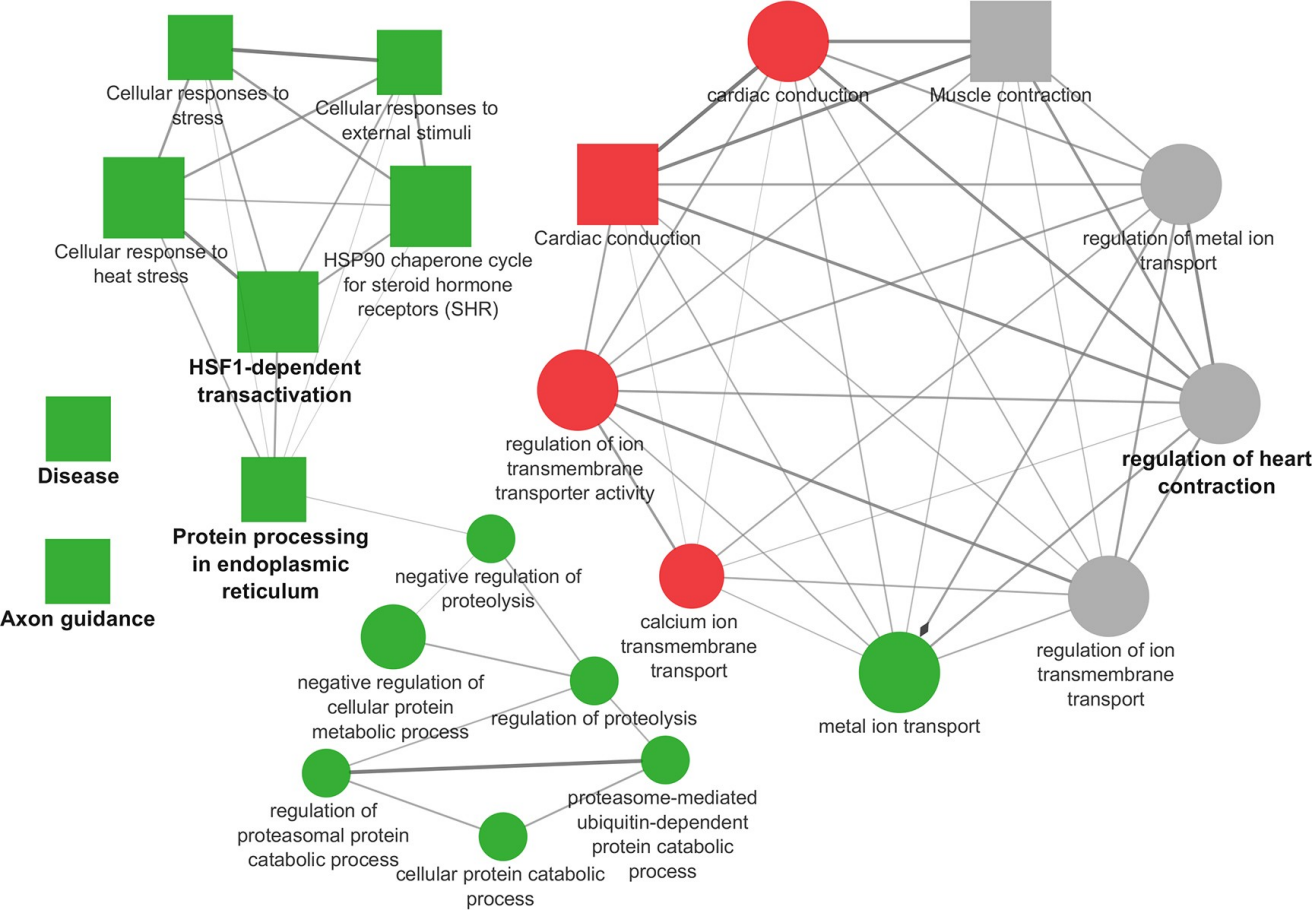
Cytoscape functional analysis of LES *vs* LPE comparison. squares = pathways; circles = biological processes; shape size = according to the *P*-value of the term in its own group; red colour = up-regulated (*cluster #1*); green colour = down-regulated (*cluster #2*); grey colour = same number of up- and down-regulated genes (*none specific cluster*); font size = according to the *P*-value of the term in its own group; interaction line thickness = according to Kappa Score value, represents the strength of the interactions, lighter colour corresponds to a lower strength while darker colour to a higher strength.

**Fig 5:**
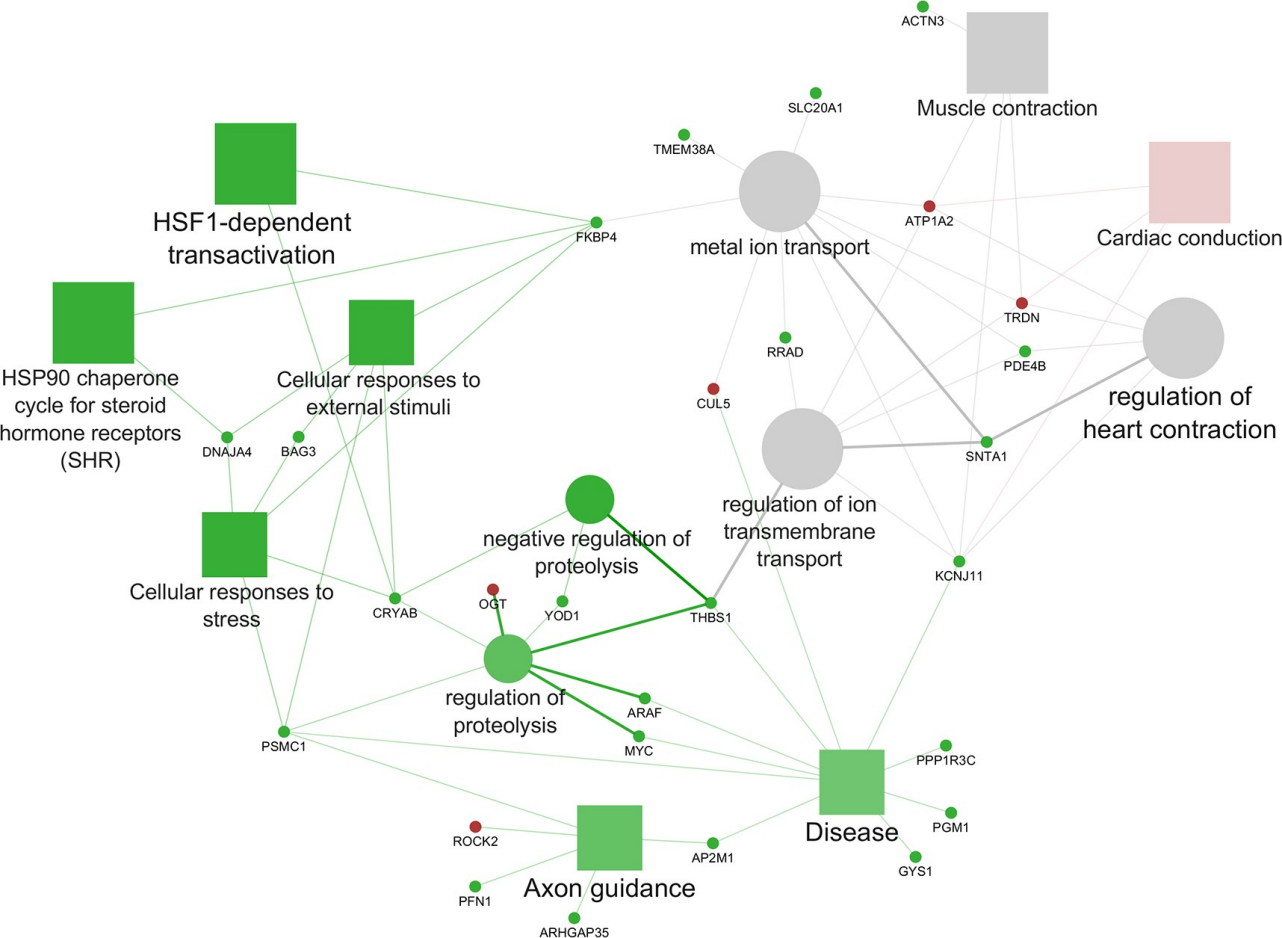
Cytoscape functional analysis of LES *vs* LPE comparison displaying hub DEGs. Significant GO terms are graphically summarized using REVIGO. squares = pathways; circles = biological processes; shape size = according to the *P*-value of the term in its own group; red colour = up-regulated (*cluster #1*); green colour = down-regulated (*cluster #2*); grey colour = same number of up- and down-regulated genes (*None specific cluster*); fill colour transparency = according to the percentage of genes belonging to the term, lighter colour corresponds to a lower percentage while darker colour to a higher percentage; font size = according to the *P*-value of the term in its own group; interaction line thickness = according to Kappa Score value, represents the strength of the interactions, lighter colour corresponds to a lower strength while darker colour to a higher strength.

In the comparison LES *vs* LPE the enriched pathways and biological processes included in the *cluster #1* with the majority of DEGs up-regulated in LES, resulted in one functional group (Figs 4 and 5, S4 Table) in which the most significant leading term was “Cardiac conduction” (*P* = 3.18E-06). The enriched pathways and biological processes included in the *cluster #2* are included in five functional groups (Figs 4 and 5, S4 Table) summarized in the following leading term: “Metal ion transport” (*P* = 0.00007); “Protein processing in endoplasmic reticulum” (*P* = 0.001), “HSF1-dependent transactivation” (*P* **=** 1.19E-06)”; “Axon guidance” (*P* = 0.0043); “Disease” (*P* = 0.0024). Finally, the enriched pathways and biological processes included in *none specific cluster* were represented by one functional group (Figs 4 and 5, S4 Table) in which the leading term was: “Regulation of heart contraction” (*P* = 1.79E-06).

### Functional analysis with DAVID

Functional analysis performed using DAVID was done to compare the results from Cytoscape analysis with another bioinformatics tool frequently used in literature. DAVID results for the comparison L *vs* LPE are displayed in Table 3. The results confirmed the majority of the pathways and biological processes found by Cytoscape in the comparison L *vs* LPE namely “Spliceosome”, “mRNA processing”, “RNA splicing”, “Focal adhesion”, “HIF-1 signaling pathway”, “Proteoglycans in cancer”, “Platelet degranulation”.

**Table 3:** DAVID functional annotation results of L *vs* LPE comparison. Only significant functional terms (*P* ≤ 0.05) are reported with the relative list of annotated genes found in this comparison.

**Category**	**Up-regulated DEGs in L compared to LPE**	***P*-value**
KEGG Pathway (201 DEGs found*)		
Spliceosome	*SRSF1*, *SRSF10*, *TRA2A*, *U2SURP*, *PRPF3*, *DDX5*, *SF3B1*, *SRSF2*, *SRSF5*, *SRSF6*, *CDC40*, *LSM5*, *THOC2*, *RBM25*	0.0241
GO Biological Process (515 DEGs found*)		
GO:0006397 mRNA processing	*SRSF1*, *PAN2*, *PAN3*, *SREK1*, *FMR1*, *SRSF11*, *PRPF3*, *MBNL1*, *NSRP1*, *SRSF2*, *SRSF5*, *SCAF11*, *PAPD4*, *CNOT6L*, *ZRANB2*, *LSM5*, *QKI*, *SREK1IP1*, *RBM25*, *RBM26*	0.0051
GO:0008380 RNA splicing	*SREK1*, *FMR1*, *SRSF11*, *MPHOSPH10*, *RBM5*, *PRPF3*, *MBNL1*, *NSRP1*, *SRSF2*, *SCAF11*, *PPIG*, *CDC40*, *ZRANB2*, *QKI*, *RNPC3*, *SREK1IP1*, *THOC2*, *RBM25*, *LUC7L3*	0.0034
GO:0006405 RNA export from nucleus	*SRSF1*, *SRSF2*, *SRSF5*, *SRSF6*, *CDC40*, *SRSF11*, *ZC3H11A*, *NXF1*, *TPR*, *THOC2*	0.0260
**Category**	**Down-regulated DEGs in L compared to LPE**	***P*-value**
KEGG Pathway (235 DEGs found*)		
Focal adhesion	*TLN2*, *BCAR1*, *MYLK2*, *ARHGAP35*, *ACTN3*, *CAPN2*, *FLNB*, *FLNA*, *COL5A1*, *ITGA5*, *MAPK3*, *COL1A2*, *COL6A2*, *COL6A1*, *COL1A1*, *ZYX*, *THBS1*, *RAPGEF1*, *THBS4*	0.0440
Insulin signaling pathway	*MAP2K2*, *FLOT2*, *HK1*, *FBP2*, *PPARGC1A*, *PRKAR2A*, *PPP1R3C*, *PYGM*, *PYGL*, *ARAF*, *MAPK3*, *GYS1*, *PRKACA*, *MTOR*, *RAPGEF1*	0.0250
Glycolysis / Gluconeogenesis	*PKM*, *GPI*, *LDHA*, *TPI1*, *PGM1*, *PGAM2*, *HK1*, *FBP2*, *GAPDH*, *ENO1*	0.0290
HIF-1 signaling pathway	*CDKN1A*, *LTBR*, *MAP2K2*, *MAPK3*, *SERPINE1*, *EGLN3*, *HK1*, *MTOR*, *GAPDH*, *STAT3*, *TIMP1*, *ENO1*	0.0250
Gap junction	*TUBB*, *ADCY1*, *TUBA8*, *GNAI2*, *MAP2K2*, *MAPK3*, *TUBA4A*, *TUBB6*, *PRKACA*, *TUBA1A*, *TUBB4B*	0.0330
Proteoglycans in cancer	*MAP2K2*, *IGF2*, *MMP2*, *FLNB*, *STAT3*, *FLNA*, *EIF4B*, *CDKN1A*, *CD44*, *ITGA5*, *ARAF*, *MAPK3*, *PRKACA*, *MTOR*, *MSN*, *THBS1*, *MYC*	0.0440
GO Biological Process (445 DEGs found*)		
GO:0006936 muscle contraction	*ACTA1*, *CRYAB*, *ACTA2*, *ACTN3*, *CACNG1*, *KCNJ12*, *CACNA1S*, *MYH8*, *DYSF*, *MYOM2*, *RYR1*, *MYOM1*, *LMOD2*, *EMD*, *SGCA*, *SNTA1*	0.0003
GO:0098609 cell-cell adhesion	*HDLBP*, *LDHA*, *TAGLN2*, *GPRC5A*, *CAPZB*, *PKM*, *PFN1*, *BAG3*, *SND1*, *RAB11B*, *HSPA5*, *EHD1*, *EMD*, *ENO1*, *EHD4*, *PLEC*, *DAB2IP*, *EEF2*, *FLNB*, *ANXA2*, *MICALL1*, *LASP1*, *UBAP2*, *EPN2*, *ADD1*	0.0003
GO:0002576 platelet degranulation	*CD9*, *SELP*, *CYB5R1*, *PSAP*, *CLU*, *SERPINE1*, *TUBA4A*, *IGF2*, *SERPING1*, *WDR1*, *THBS1*, *ECM1*, *FLNA*, *TIMP1*	0.0025
GO:0006094 gluconeogenesis	*GPI*, *TPI1*, *ATF3*, *PGM1*, *PGAM2*, *FBP2*, *PPARGC1A*, *GAPDH*, *ENO1*	0.0100
GO:0006096 glycolytic process	*GPI*, *LDHA*, *TPI1*, *PGM1*, *PGAM2*, *HK1*, *GAPDH*, *ENO1*	0.0120
GO:0007155 cell adhesion	*TLN2*, *BCAR1*, *BCAM*, *NEO1*, *CD151*, *CD9*, *CD44*, *TGFBI*, *COL6A2*, *COL6A1*, *ZYX*, *THBS1*, *THBS4*, *ICAM1*, *SYMPK*, *SELP*, *FLOT2*, *ADGRE5*, *MFGE8*, *CTNNA1*, *COL5A1*, *THY1*, *CDH13*, *RND3*, *ITGA5*, *COL1A1*, *SEMA4D*, *ENG*, *EMP2*	0.0150
GO:0061621 canonical glycolysis	*PKM*, *GPI*, *TPI1*, *PGAM2*, *HK1*, *GAPDH*, *ENO1*	0.0160
GO:0008219 cell death	*FOSL2*, *HMOX1*, *CLU*, *RRAGA*, *EMP3*, *PMP22*, *EMP2*, *EMP1*	0.0190
GO:0036498 IRE1-mediated unfolded protein response	*XBP1*, *TPP1*, *LMNA*, *SRPRA*, *HSPA5*, *ASNA1*, *DCTN1*, *SEC61A2*, *ADD1*	0.0390
GO:0071230 cellular response to amino acid stimulus	*XBP1*, *COL1A2*, *RRAGA*, *COL6A1*, *COL1A1*, *CAPN2*, *MMP2*, *NEURL1*	0.0500

* = Number of DEGs found associated within each category (GO Biological Process or Pathway).

The LES *vs* LPE results are showed in Table 4, and also in this comparison, some results are similar to those from Cytoscape, namely “Regulation of cardiac conduction” and “Regulation of cellular response to heat”. However, the number of DEGs included by DAVID software in each pathway or biological process was lower than the respective one using Cytoscape. The reason for the different number of DEGs clustered in each function by the two software is likely attributable to differences in the statistical methods used and to the different setting tools. Overall, even considering these differences, the pathways and BPs reported by the two software were concordant.

**Table 4:** DAVID functional annotation results of LES *vs* LPE comparison. Only significant functional terms (*P* ≤ 0.05) are reported with the relative list of annotated genes found in this comparison.

**Category**	**Up-regulated DEGs in LES compared to LPE**	***P*-value**
KEGG Pathway (9 DEGs found*)		
cGMP-PKG signaling pathway	*ATP1B1*, *ROCK2*, *PLN*, *ATP1A2*	0.0236
cAMP signaling pathway	*ATP1B1*, *ROCK2*, *PLN*, *ATP1A2*	0.0228
GO Biological Process (17 DEGs found*)		
GO:1903779 regulation of cardiac conduction	*TRDN*, *ATP1B1*, *PLN*, *ATP1A2*	0.0041
GO:0055119 relaxation of cardiac muscle	*ATP1B1*, *PLN*, *ATP1A2*	0.0060
**Category**	**Down-regulated DEGs in LES compared to LPE**	***P*-value**
GO Biological Process (57 DEGs found*)		
GO:0006457 protein folding	*HSP90AB1*, *HSP90AA1*, *CRYAB*, *FKBP4*, *BAG3*, *SACS*, *DNAJA4*, *HSPA8*	0.0014
GO:1900034 regulation of cellular response to heat	*HSP90AB1*, *HSP90AA1*, *CRYAB*, *FKBP4*, *BAG3*, *HSPA8*	0.0016

* = Number of DEGs found associated with each category (GO Biological Process or Pathway).

### Validation using RT-qPCR

The validation of RNA-Seq results by RT-qPCR showed agreement between the gene expression data obtained with the two methods. The five genes validated showed an overall good correlation between the two methods (Table 5).

**Table 5:** Pearson’s correlation coefficient (r) and *P*-values between RNA-Seq and RT-qPCR expression values of five selected genes.

Gene	r coefficient	*P*-value	Diet comparison^(1)^
***PPARGC1A***	0.77	< 0.0001	L-LPE
***THBS1***	0.73	< 0.0001	LES-LPE
***THBS1***	0.56	0.006	L-LPE
***TGFBI***	0.53	0.008	L-LPE
***LPL***	0.52	0.01	L-LPE
***RXRA***	0.44	0.04	L-LPE

^(1)^L = diet enriched with extruded linseed (source of *n-*3 PUFA); LES = diet enriched with extruded linseed, vitamin E and selenium; LPE = diet enriched with extruded linseed and plant extracts (source of polyphenols).

## Discussion

Over the last two decades, there has been an increasing interest in the beneficial effects of bioactive lipids on human health. Among them, a large body of evidence indicated the prominent role of *n*-3 PUFA as protective lipid mediators in the context of inflammatory, metabolic, neurodegenerative, and neoplastic diseases [33,34]. This evidence has drawn the attention of clinicians and animal scientists to the possibility of turning animal-derived products into functional foods providing health benefits to consumers [35–37]. Studies have reported that the content of *n*-3 PUFA in swine muscle can be increased adding oilseed ingredients to the feed [38,39], and the addition of antioxidant compounds such as vitamin E and polyphenols can help to avoid lipid oxidation [38,40]. Anyway, the knowledge on the muscle physiological processes influenced by dietary antioxidant addition is still poor and could involve a large number of genes associated with both muscle metabolism and structure [41]. Transcriptomics actually represents one of the most relevant techniques in nutrigenomics studies to obtain a comprehensive analysis of gene expression changes and molecular processes influenced by the diet components in order to increase the scientific understanding of the biology behind production traits [41,42].

In L *vs* LPE comparison the majority of the hub genes displayed in Fig 3 was related to “RNA metabolic process”, a large GO term including cellular processes involving RNA and linked to “mRNA metabolic process”, “RNA splicing” and “negative regulation of protein metabolic process”. Interestingly, the percentages of genes up- and down-regulated by the two diets are very similar (S4 Table), thus suggesting that both diets may overall influence the transcriptional activity and the splicing of muscle mRNA. Indeed, recent studies have reported that macro- and micronutrients, including PUFA and resveratrol, could influence the regulation of pre-mRNA splicing and in particular can modulate the expression of the Serine/Arginine-rich protein gene family (*SRs*) and *HNRNP1*, like in our study, that in turns regulate the alternative splicing of several genes involved in lipid and energy metabolism [43,44]. In L *vs* LPE, among the DEGs, one of the most interesting hub genes linking “RNA metabolic process”, “mRNA metabolic process”, “RNA splicing” and “negative regulation of protein metabolic process”, is *PPARGC1A* (also known as *PGC-1α*), that is up-regulated in LPE compared to L. This gene is a member of PPARGs family and it is a transcriptional coactivator of *PPARG*. It is well known from *in vitro* studies that the expression of *PPARG*, and more generally of the PPARGs family, can be stimulated by both dietary *n-*3 PUFA and polyphenols [45]. This statement is in agreement with our results since we observed an up-regulation of *PPARGC1A* in LPE group. In literature, it is reported that *PPARGC1A* may be involved in many biological mechanisms like fatty acid oxidation, glucose utilization, mitochondrial biogenesis, angiogenesis and muscle trophic stimulation [46]. Recent literature [47] demonstrated the role of *PPARGC1A* in the maintenance of lipid balance *via* its engagement in numerous metabolic processes related to lipid synthesis and/or lipid utilization (*i*.*e*. Krebs cycle, β-oxidation, oxidative phosphorylation and electron transport chain). In particular, in insulin-sensitive tissues such as the skeletal muscle, *PPARGC1A* is regarded as one of the factors eliciting FA uptake by the cells, controlling their oxidation for energy purposes or esterification for the conversion into bioactive fractions [47]. Other regulatory genes up-regulated in LPE compared to L are *PRKACA* and *PRKAR2A*, two genes also involved in glucose and lipid homeostasis and in cell growth [48,49]. The low number of DEGs related to lipid metabolism found in this comparison can be explained by the fact that all the diets had the same amount of extruded linseed as a source of *n*-3 PUFA. Therefore the differences observed in the expression of genes with a role in lipid metabolism are reasonably due to the concurrent polyphenols supplementation and/or to their interaction with the other components of the diet.

The results obtained with DAVID showed that *PPARGC1A*, *PRKACA* and *PRKAR2* were also involved in glucose and glycogen metabolism and in the insulin signaling pathway, together with other DEGs found up-regulated in LPE, namely *ARAF*, *FBP2*, *FLOT2*, *GYS1*, *HK1*, *MAPK2*, *MAPK3*, *MTOR*, *PPP1R3C*, *PYGL*, *PYGM*, *RAPGEF*. Moreover, our results showed also the up-regulation in LPE, of some genes involved in “Translocation of GLUT4 to the plasma membrane” (*MYH8*, *MYO1C*, *RALGAPA2*, *TUBA1A*, *TUBA4A*, *TUBA8*, *TUBB4B*, *TUBB6*). On the whole, considering these results, it could be postulated that in LPE group the diet has induced an increased glucose uptake by the muscle cells. Indeed, it is worth noting that glucose uptake is endorsed by the translocation of GLUT4 vesicles to the plasma membrane [50] and accordingly, the capacity of polyphenols to enhance glucose uptake was already reviewed in muscle cells [51,52]. An increased glucose uptake was also found to stimulate *PRKAR2* expression in human muscle [53]. Therefore the increased modulation of functions related to glucose metabolism, observed in LPE compared to L in the present study, seems to be mainly ascribable to polyphenols supplementation, according to previous literature findings, reporting that polyphenols can up-regulate the expression of genes involved in the insulin signaling pathway and in glucose uptake by the cells [54–56].

Many DEGs involved in glucose and glycogen metabolism can play also a role in the “HIF-1 signaling pathway”. For example, glucose-dependent insulin release in response to feeding was found to increase *MTOR* expression which may enhance energy storage and growth in the muscle tissue [57]. Other genes of the HIF-1 signaling pathway with a role in glucose metabolism, were also *ENO1*, *GAPDH*, *HK1*, *LDHA*, and the transcriptional factors *MAPK2* and *MAPK3*. These genes were all up-regulated in LPE compared to L. In particular, it was reported that the up-regulation of *MAPK* and *MTOR* can activate the HIF-1 signaling pathway [58,59] and plays a pivotal role in the regulation of oxygen and glucose cellular utilization, promoting anaerobic glycolysis. The overexpression of *LDHA* in the LPE individuals seems, therefore, to confirm the presence of anaerobic glycolysis processes in the muscle since in literature the overexpression of *LDHA* was found associated with the utilization of glucose in the anaerobic metabolism and with the regulation of lactate homeostasis in pig cardiac muscle [51]. In this view, in the present study, the up-regulation of HIF-1 signaling pathway, in pigs fed LPE diet, seems to reveal the presence of anaerobic glycolysis addressed to energy production in the myocytes. Anyway, this hypothesis would need to be validated with phenotypic measures on muscle tissue. Moreover, the HIF-1 signaling pathway is also involved in the control of cellular growth and proliferation. DEGs with a role in cellular proliferation were *CDKN1A*, *LTBR* and the transcriptional factors *MAPK* and *MTOR*, up-regulated in LPE. Actually, some papers [57,60] reported that when *MTOR* is up-regulated with *AKT* (as suggested by the similar expression pattern found in the present study) it may stimulate the expression of genes involved in skeletal muscle tissue trophy. Considering, therefore, the concurrent up-regulation of the *MAPK2*, *MAPK3*, *MTOR*, and *STAT3* genes in the LPE dietary group, it is possible to presume an effect of the LPE diet in favouring muscle cells growth by stimulating the HIF-1 signaling pathway. Indeed, the present research also showed that LPE diet compared to L diet up-regulated many genes clustered in the “Muscle organ development” BP (*ACTA1*, *ACTN3*, *ATF3*, *BTG2*, *BVES*, *CASQ1*, *EEF2*, *EMD*, *FLNB*, *MAFF*, *MEF2D*, *MYH14*, *MYLK2*, *MYOM1*, *MYOM2*, *NEURL1*, *RYR1*, *SELENON*, *SMYD1*). This result enforces the hypothesis that a diet supplemented with *n*-3 PUFA and polyphenols may enhance muscle growth. In addition, among the above-cited genes, the hub gene *BTG2* is an important regulatory element that belongs to the anti-proliferative family genes playing an important role in the regulation of the transcriptional activity and in tumour suppression [61]. In studies on lambs and growing pigs [62,63], *BTG2* was reported to be highly expressed in skeletal muscle tissue and its overexpression was associated with increased muscle cells size thus promoting muscle hypertrophy. Of particular interest is the study of Mo *et al*. [62] who reported, in two swine breeds, a higher expression of *BTG2* in the muscle of lean-type pigs in comparison to the fat-type breed. Interestingly, in human oncological studies, the expression of *BTG2* was found stimulated by dietary polyphenols [64,65], supporting the hypothesis of an increased expression of *BTG2* as an effect of LPE diet in the present study.

Lastly, the results from the present study evidenced also a relationship among “HIF-1 signaling pathway” and “Platelet activation, signaling and aggregation” and “Hemostasis” through the hub genes *TIMP1* and *ECM1*, up-regulated in LPE compared to L (Fig 3). Other genes in these pathways, also up-regulated in LPE compared to L, are *THBS1* and *SERPINE1*. These genes, together with *TIMP1*, are known to have a role as anti-angiogenic factors that may be released by platelet activation [66]. Indeed, platelets can release pro- and anti-angiogenic proteins that regulate angiogenetic processes [66]. The release of these proteins as result of platelet activation is involved in vascular development. Moreover, compared to L, the LPE diet showed to down-regulate *VEGF* and *PI3K*, which are key genes involved in angiogenesis, thus supporting also an effect of polyphenols and *n*-3 PUFA in inhibiting angiogenesis in agreement with data reported in the literature [67–69]. By contrast *IGF2*, a gene coding for a pro-angiogenic factor was also up-regulated in LPE. This result is not surprising because in physiological conditions platelets fulfil, through the balanced release of pro- and anti-angiogenic proteins, many key roles in establishing and maintaining vascular homeostasis and vascular integrity in all the tissues [66].

Overall, the results obtained in L *vs* LPE comparison suggested that plant extracts added to *n*-3 PUFA in LPE increased the expression of genes involved in the stimulation of mRNA metabolic process addressed to glucose uptake and cells growth in pig muscle tissue. Further comparison with the literature is difficult or not reliable because most of the published studies assessed the effect of *n*-3 PUFA and polyphenols on gene expression under pathological conditions (*e*.*g*. obesity, diabetes, tumour, ageing and neurodegenerative disease) and in different species. Therefore, it is important to report that only a few studies were found aimed to analyse the effect of these compounds on the transcriptome of healthy growing animals, and in livestock species. Among the few papers evaluating the effects of polyphenols and *n-*3 PUFA on the mRNA and protein levels of genes in skeletal muscle, the one from Gutierrez-Salmean *et al*. [70] reported that the treatment with epicatechin (belonging to the flavonols class) positively increased the amounts of proteins encoded by genes involved in the regulation of skeletal muscle growth and differentiation. Moreover, Kaminski *et al*. [71] found that resveratrol, a grape-derived polyphenolic compound, is able to upregulate the expression of genes involved in the control of metabolic pathways in mouse skeletal muscle cells, thus supporting a positive effect of this compound on skeletal muscle function. Recently, Dugdale *et al*. [72] have evidenced that resveratrol has also the potential to maintain appropriate muscle cell functions, stimulating muscle cell growth and regeneration. Also, *n-*3 PUFA has been implicated in improving muscle efficiency and may contribute to maintaining both muscle mass and function [73–75]. Unless the result of the present research need to be further validated by phenotypic traits, comparison with the found literature allows to hypothesize that, considering the upregulation in LPE of several genes encoding for proteins involved in muscle development and functions, the combined supplementation with polyphenols and *n-*3 PUFA can enhance skeletal muscle efficiency and structure, compared to the only *n-*3 PUFA supplementation in L.

The LES *vs* LPE comparison showed that the diet supplemented with *n-*3 PUFA, vitamin E and selenium (LES) reduced the expression of several genes, while the diet supplemented with *n-*3 PUFA and plant extracts (LPE) showed the general effect of stimulating gene expression. As previously reported in the results section of the manuscript, it is possible to observe that 70 out of 80 DEGs (Fig 1) in LES *vs* LPE comparison are the same found differentially expressed in L *vs* LPE also showing the same expression trend. This result suggests that the diet added with vitamin E appears to have similar but slighter effects on pig transcriptome, compared to the diet added with plant extracts. The ten genes left, *DMPK*, *DNAJA4*, *HSPA8*, *HSP90AA1*, *HSP90AB1*, *IER5*, *PDE4B*, *PSMC1*, *SLC20A1*, *STIP1*, were not shared between the two comparisons but they were also involved in functions related to those previously found and described in L *vs* LPE comparison. In fact, *DMPK* is a gene influencing the maintenance of functions and structure of the skeletal muscle [76] and *DNAJA4*, *HSPA8*, *HSP90AA1*, *HSP90AB1*, *IER5*, *PDE4B*, *STIP1* are all involved in cellular signaling and in response to cellular stress [77–83].

Therefore, the main differences between LPE and LES are referred to the level of transcription of some genes up-regulated in LPE (*BAG3*, *CRYAB*, *DNAJA4*, *FKBP4*, *HSP90AA1*, *HSP90AB1*, *HSPA8*, *PSMC1*, *STIP1*) and involved in pathways linked to the cellular stress response such as “HSF1-dependent transactivation”, “HSP90 chaperone cycle for steroid hormone receptors (SHR)”, “Cellular responses to external stimuli” and “Cellular responses to stress”. These pathways were connected through some hub genes (*BAG3*, *CRYAB*, *DNAJA4*, *PSMC1*) to the BPs involved in protein metabolism such as “Negative regulation of proteolysis” and “Regulation of proteolysis”. It is worth noting that these genes (especially the members of the HSPs family) are not only involved in the cellular stress response but also in basic cellular processes related to protein folding and trafficking, addressed to maintain and support physiological muscle activity [84,85]. There is a lack of studies on the effects of polyphenols on these genes, in particular about the role of these bioactive compounds on the skeletal muscle physiology of healthy individuals, like in the present research. Other DEGs between LES and LPE are involved in functions related to the muscle contraction and conduction such as “Metal ion transport”, “regulation of ion transmembrane transport”, “Muscle contraction”, “Regulation of heart contraction” and “Cardiac conduction”, which are all connected through the hub genes *THBS1* and *FKBP4*. These pathways and BPs related to muscle contraction, contained in similar proportion up-regulated genes (*AKAP9*, *ATP1A2*, *ATP1B1*, *CUL5*, *PLN*, *TRDN*) and down-regulated genes (*ATP1A1*, *DMPK*, *FKBP4*, *KCNJ11*, *PDE4B*, *RRAD*, *SLC20A1*, *SNTA1*, *TMEM38A*) in LES compared to LPE. In fact, in the present study, some genes coding for proteins involved in ion transmembrane transport and calcium-release complex addressed to muscle contraction and relaxation (*ATP1A1*, *KCNJ11*, *DMPK*, *RRAD*, *TMEM38A*), were found up-regulated in LPE compared to LES. Also, this result leads to support, in the present study, a stronger effect of polyphenols in improving muscle functionality compared to the vitamin E supplementation. However, the knowledge about the effects of plant-derived polyphenols is still very limited in livestock species and sometimes the results are controversial, because of substantial differences among the experimental conditions [18].

Interestingly, in LES *vs* LPE comparison, we did not find any DEGs among those that in L *vs* LPE were involved in the “HIF-1 signaling pathway”. Since the genes of the “HIF-1 signaling pathway” were either not differentially expressed in L *vs* LES, it is possible to hypothesize that the effect of the diet integration on gene expression was of a lesser extent in LES (*i*.*e*. the transcript levels of the genes involved in HIF-1 signaling pathway were in-between the lowest transcriptional level found in L group and the highest expression found for the same genes in LPE dietary group). Therefore, it is possible to hypothesise that the addition of vitamin E and Selenium to a diet rich in *n*-3 PUFA (LES) caused, in the present study, a low-magnitude effect on swine skeletal muscle transcriptome compared to plant-derived polyphenols (LPE). The different effect observed between the two groups fed antioxidant compounds (LES and LPE) should be imputable to the composition of the antioxidant integration (vitamin E or plant-derived polyphenols). In fact, it has been reviewed that a mixture of polyphenolic compounds may have a greater phenotypic effect on a tissue compared to the administration of a single compound [86] unless other studies reported opposite results [87]. Moreover, it is important to consider that many other factors could have been occurred, since the knowledge on the *in vivo* molecular effects of polyphenols and antioxidant compounds is presently scant in the livestock species, as well as the knowledge on the bioavailability and on the interaction among plant extracts and nutrients in the diet [88]. To the best of Authors’ knowledge, this is one of the few studies investigating the *in vivo* effects of dietary *n-*3 PUFA and antioxidants on the transcriptome of *Longissimus thoracis* in swine, and the results need to be further elucidated considering potential phenotypic effects in the porcine muscle tissue.

## Conclusions

The present study evidenced that adding antioxidants to a *n*-3 PUFA-rich diet can influence the transcription level of *Longissimus thoracis* muscle in pigs. These effects were more evident with the addition of plant extracts (source of polyphenols) compared to the addition of vitamin E. Indeed, the diet with linseed and plant extracts showed to up-regulate a large number of genes compared to the linseed supplementation alone. These genes were involved in many functions belonging to the regulation of transcriptional activity and glucose metabolism aimed to support muscle tissue trophy, vascular homeostasis and muscle activity. Differently, the addition of vitamin E to a *n*-3 PUFA-rich diet did not show any significant difference in the transcriptome compared to the linseed supplementation alone. These findings provide new knowledge on the effects of dietary plant extracts and *n*-3 PUFA on pig muscle transcriptome. Further investigation on the phenotypical effects *in vivo* and in derived meat products, as well as the potential benefits for consumers, are needed for both research and industry purposes.

## Supporting information

S1 Table List of the genes and primers tested by RT-qPCR for validation. (DOCX)

S2 Table Complete list of reads obtained from RNA-Seq analysis. (XLSX)

S3 Table Complete list of differentially expressed genes in each diet comparison. (XLSX)

S4 Table Full results from Cytoscape functional analysis. (XLS)

## References

[1] WHO 2003. Diet, Nutrition and the Prevention of Report of a Joint WHO / FAO Expert Consultation 2003; 1–149.

[2] Wood JD, Richardson RI, Nute GR, Fisher A V., Campo MM, Kasapidou E, et al Effects of fatty acids on meat quality: A review. Meat Sci. 2004;66: 21–32. 10.1016/S0309-1740(03)00022-6 22063928

[3] Lahučký R., Bahelka I., Novotná K.& Vašíčková K. Effects of dietary vitamin E and vitamin C supplementation on the level of α-tocopherol and L-ascorbic acid in muscle and on the antioxidative status and meat quality of pigs. Czech J Anim Sci. 2005;50: 175–184.

[4] Niculita P, Popa ME, Ghidurus M, Turtoi M. Effect of vitamin e in swine diet on animal growth performance and meat quality parameters. Polish J Food Nutr Sci. 2007;57: 125–130.

[5] Kawęcka M, Jacyno E, Matysiak B, Kołodziej-Skalska A, Pietruszka A. Effects of selenium and vitamin E supplementation on selenium distribution and meat quality of pigs. Acta Agric Scand 2013;63: 194–200.

[6] Morel PCH, Leong J, Nuijten WGM, Purchas RW, Wilkinson BHP. Effect of lipid type on growth performance, meat quality and the content of long chain n − 3 fatty acids in pork meat. Meat Sci. 2013; 95: 151–159. 10.1016/j.meatsci.2013.04.047 23739265

[7] Pieszka M, Szczurek P, Bederska-Łojewska D, Migdał W, Pieszka M, Gogol P, et al The effect of dietary supplementation with dried fruit and vegetable pomaces on production parameters and meat quality in fattening pigs. Meat Sci. 2017;126: 1–10. 10.1016/j.meatsci.2016.11.016 27978462

[8] Oczkowicz M., Świątkiewicz M., Ropka-Molik K., Gurgul A. & Żukowski K. (2016) Effects of different sources of fat in the diet of pigs on the liver transcriptome estimated by RNA-seq. Ann of Anim Sci. 2016;16: 1073–1090.

[9] Szostak A, Ogłuszka M, te Pas MFW, Poławska E, Urbański P, Juszczuk-Kubiak E, et al Effect of a diet enriched with omega-6 and omega-3 fatty acids on the pig liver transcriptome. Genes Nutr. 2016;11: 9 10.1186/s12263-016-0517-4 27482299 PMC4959555

[10] Azorín-Ortuño M, Yáñez-Gascón MJ, González-Sarrías A, Larrosa M, Vallejo F, Pallarés FJ, et al Effects of long-term consumption of low doses of resveratrol on diet-induced mild hypercholesterolemia in pigs: A transcriptomic approach to disease prevention. J Nutr Biochem. 2012;23: 829–837. 10.1016/j.jnutbio.2011.04.007 21852083

[11] Fiesel A, Gessner DK, Most E, Eder K. Effects of dietary polyphenol-rich plant products from grape or hop on pro-inflammatory gene expression in the intestine, nutrient digestibility and faecal microbiota of weaned pigs. BMC Vet Res. 2014;10: 196 10.1186/s12917-014-0196-5 25323005 PMC4158062

[12] Zhang C, Luo J, Yu B, Zheng P, Huang Z, Mao X, et al Dietary resveratrol supplementation improves meat quality of finishing pigs through changing muscle fiber characteristics and antioxidative status. Meat Sci. 2015;102: 15–21. 10.1016/j.meatsci.2014.11.014 25524822

[13] Gessner DK, Bonarius M, Most E, Fiesel A, Eder K. Effects of polyphenol-rich plant products from grape or hop as feed supplements on the expression of inflammatory, antioxidative, cytoprotective and endoplasmic reticulum stress-related genes and the antioxidative status in the liver of piglets. J Anim Physiol Anim Nutr. 2017;101: e185–e194. 10.1111/jpn.12586 27561387

[14] Liu F, Cottrell JJ, Furness JB, Rivera LR, Kelly FW, Wijesiriwardana U, et al Selenium and vitamin E together improve intestinal epithelial barrier function and alleviate oxidative stress in heat-stressed pigs. Exp Physiol. 2016;101: 801–810. 10.1113/EP085746 27064134

[15] Oh DY, Talukdar S, Bae EJ, Imamura T, Morinaga H, Fan WQ, et al GPR120 Is an Omega-3 Fatty Acid Receptor Mediating Potent Anti-inflammatory and Insulin-Sensitizing Effects. Cell. 2010;142: 687–698. 10.1016/j.cell.2010.07.041 20813258 PMC2956412

[16] De Groote D, Van Belleghem K, Devire J, Van Brussel W, Mukaneza A, Amininejad L. Effect of the intake of resveratrol, resveratrol phosphate, and catechin-rich grape seed extract on markers of oxidative stress and gene expression in adult obese subjects. Ann Nutr Metab. 2012;61: 15–24. 10.1159/000338634 22776850

[17] Hussain T., Tan B., Yin Y., Blachier F., Tossou M.C.B. and Rahu N. Oxidative stress and inflammation: what polyphenols can do for us? Oxid Med Cell Lon. 2016;2016: 7432797. 10.1155/2016/7432797 PMC5055983 27738491

[18] Kim YA, Keogh JB, Clifton PM. Polyphenols and glycémie control. Nutrients. 2016;8. 10.3390/nu8010017 PMC4728631 26742071

[19] Lipiński K, Mazur M, Antoszkiewicz Z, Purwin C. Polyphenols in monogastric nutrition—A review. Ann Anim Sci. 2017;17: 41–58.

[20] Corino C, Musella M, Mourot J. Influence of extruded linseed on growth, carcass composition, and meat quality of slaughtered pigs at one hundred ten and one hundred sixty kilograms of liveweight. J Anim Sci. Oxford University Press; 2008;86: 1850–1860. 10.2527/jas.2007-0155 18441078

[21] Lo Fiego D Pietro, Macchioni P, Minelli G, Santoro P. Lipid composition of covering and intramuscular fat in pigs at different slaughter age. Ital J Anim Sci. 2010;9: 200–205.

[22] Rossi R, Ratti S, Pastorelli G, Crotti A, Corino C. The effect of dietary vitamin E and verbascoside on meat quality and oxidative stability of Longissimus Dorsi muscle in medium-heavy pigs. Food Res Int. Elsevier; 2014;65: 88–94.

[23] Dobin A, Davis CA, Schlesinger F, Drenkow J, Zaleski C, Jha S, et al STAR: Ultrafast universal RNA-seq aligner. Bioinformatics. 2013;29: 15–21. 10.1093/bioinformatics/bts635 23104886 PMC3530905

[24] Liao Y, Smyth GK, Shi W. FeatureCounts: An efficient general purpose program for assigning sequence reads to genomic features. Bioinformatics. 2014;30: 923–930. 10.1093/bioinformatics/btt656 24227677

[25] Robinson MD, Mccarthy DJ, Smyth GK. edgeR: a Bioconductor package for differential expression analysis of digital gene expression data. Bioinformatics. 2010;26: 139–14010. 10.1093/bioinformatics/btp616 19910308 PMC2796818

[26] Tarazona S, Furió-Tarí P, Turrà D, Di Pietro A, Nueda MJ, Ferrer A, et al Data quality aware analysis of differential expression in RNA-seq with NOISeq R/Bioc package. Nucleic Acids Res. 2015;43. 1 10.1093/nar/gkv711 PMC4666377 26184878

[27] Pfaffl MW. Quantification strategies in real-time PCR In: Bustin SA, editor. A-Z of Quantitative PCR. La Jolla: International University Line (IUL); 2004 pp. 87–112.

[28] Zappaterra M, Deserti M, Mazza R, Braglia S, Zambonelli P, Davoli R. A gene and protein expression study on four porcine genes related to intramuscular fat deposition. Meat Sci. Elsevier; 2016;121: 27–32. 10.1016/j.meatsci.2016.05.007 27236338

[29] R Core Team. R: A language and environment for statistical computing. Vienna, Austria: R Foundation for Statistical Computing; 2017.

[30] Montojo J, Zuberi K, Rodriguez H, Kazi F, Wright G, Donaldson SL, et al GeneMANIA Cytoscape plugin: fast gene function predictions on the desktop. Bioinformatics. Oxford University Press; 2010;26: 2927–2928. 10.1093/bioinformatics/btq562 20926419 PMC2971582

[31] Bindea G, Mlecnik B, Hackl H, Charoentong P, Tosolini M, Kirilovsky A, et al ClueGO: a Cytoscape plug-in to decipher functionally grouped gene ontology and pathway annotation networks. Bioinformatics. 2009;25: 1091–1093. 10.1093/bioinformatics/btp101 19237447 PMC2666812

[32] Huang L, Min J-N, Masters S, Mivechi NF, Moskophidis D. Insights into function and regulation of small heat shock protein 25 (HSPB1) in a mouse model with targeted gene disruption. genesis. Wiley-Blackwell; 2007;45: 487–501. 10.1002/dvg.20319 17661394

[33] Calviello G, Su H-M, Weylandt KH, Fasano E, Serini S, Cittadini A. Experimental evidence of ω-3 polyunsaturated fatty acid modulation of inflammatory cytokines and bioactive lipid mediators: their potential role in inflammatory, neurodegenerative, and neoplastic diseases. Biomed Res Int. 2013;2013: 743171 10.1155/2013/743171 23691510 PMC3652138

[34] Calder PC. Very long-chain n-3 fatty acids and human health: fact, fiction and the future. Proc Nutr Soc. Cambridge University Press; 2018;77: 52–72. 10.1017/S0029665117003950 29039280

[35] Stankus T. Turning Meat, Poultry, Eggs, and Dairy Products Into Nutraceuticals, Part Two: The Literature of Animal Nutrition Aimed at Increasing Conjugated Linoleic Acid Levels in Beef, Lamb, Goat, Pork, and Broilers as a Part of a Value-Added Functional Foods Strategy. J Agric Food Inf. 2009;10: 37–62.

[36] Wang Y, Jacome-Sosa MM, Proctor SD. The role of ruminant trans fat as a potential nutraceutical in the prevention of cardiovascular disease. Food Res Int. 2012;46: 460–468.

[37] Mapiye C, Aalhus JL, Vahmani P, Rolland DC, McAllister TA, Block HC, et al Improving beef hamburger quality and fatty acid profiles through dietary manipulation and exploitation of fat depot heterogeneity. J Anim Sci Biotechnol. 2014;5: 54 10.1186/2049-1891-5-54 25810905 PMC4373243

[38] Buckley DJ, Morrissey PA, Gray JI. Influence of dietary vitamin E on the oxidative stability and quality of pig meat. J Anim Sci. 1995;73: 3122–3130. 8617685 10.2527/1995.73103122x

[39] Ma X, Jiang Z, Lai C. Significance of Increasing n-3 PUFA Content in Pork on Human Health. Crit Rev Food Sci Nutr. 2016;56: 858–870. 10.1080/10408398.2013.850059 26237277

[40] Cimmino R, Barone CMA, Claps S, Varricchio E, Rufrano D, Caroprese M, et al Effects of dietary supplementation with polyphenols on meat quality in Saanen goat kids. BMC Vet Res. 2018;14: 181 10.1186/s12917-018-1513-1 29890971 PMC5996534

[41] Guo B, Dalrymple BP. Transcriptomics of Meat Quality. New Aspects of Meat Quality. Woodhead Publishing; 2017 pp. 259–320.

[42] Andersen HJ, Oksbjerg N, Young JF, Therkildsen M. Feeding and meat quality—A future approach. Meat Sci. 2005;70: 543–554. 10.1016/j.meatsci.2004.07.015 22063752

[43] Ravi S, Schilder RJ, Kimball SR. Role of precursor mRNA splicing in nutrient-induced alterations in gene expression and metabolism. J Nutr. 2015;145: 841–846. 10.3945/jn.114.203216 25761502 PMC4408736

[44] Markus MA, Marques FZ, Morris BJ. Resveratrol, by Modulating RNA Processing Factor Levels, Can Influence the Alternative Splicing of Pre-mRNAs. PLoS One. 2011;6: e28926 10.1371/journal.pone.0028926 22174926 PMC3236773

[45] El Akoum S. PPAR Gamma at the Crossroads of Health and Disease: A Masterchef in Metabolic Homeostasis. Endocrinol Metab Syndr. 2014;3: 1–12.

[46] Ahmetov II, Fedotovskaya ON. Current Progress in Sports Genomics. Adv Clin Chem. 2015;70: 247–314. 10.1016/bs.acc.2015.03.003 26231489

[47] Supruniuk E, Mikłosz A, Chabowski A. The Implication of PGC-1α on Fatty Acid Transport across Plasma and Mitochondrial Membranes in the Insulin Sensitive Tissues. Front Physiol. 2017;8: 923 10.3389/fphys.2017.00923 29187824 PMC5694779

[48] Turnham RE, Scott JD. Protein kinase A catalytic subunit isoform PRKACA; History, function and physiology. Gene. 2016;577: 101–108. 10.1016/j.gene.2015.11.052 26687711 PMC4713328

[49] Park JC, Kim SC, Lee SD, Jang HC, Kim NK, Lee SH, et al Effects of dietary fat types on growth performance, pork quality, and gene expression in growing-finishing pigs. Asian-Australasian J Anim Sci. 2012;25: 1759–1767. 10.5713/ajas.2012.12416 PMC4094162 25049542

[50] Fletcher LM, Welsh GI, Oatey PB, Tavaré JM. Role for the microtubule cytoskeleton in GLUT4 vesicle trafficking and in the regulation of insulin-stimulated glucose uptake. Biochem J. 2000;352: 267–276. 11085918 PMC1221456

[51] Qiu H, Xu X, Fan B, Rothschild MF, Martin Y, Liu B. Investigation of LDHA and COPB1 as candidate genes for muscle development in the MYOD1 region of pig chromosome 2. Mol Biol Rep. 2010;37: 629–636. 10.1007/s11033-009-9882-y 19830590

[52] Hanhineva K, Törrönen R, Bondia-Pons I, Pekkinen J, Kolehmainen M, Mykkänen H, et al Impact of dietary polyphenols on carbohydrate metabolism. International Journal of Molecular Sciences. 2010;11: 1365–1402. 10.3390/ijms11041365 20480025 PMC2871121

[53] Marouli E, Kanoni S, Dimitriou M, Kolovou G, Deloukas P, Dedoussis G. Lifestyle may modify the glucose-raising effect of genetic loci. A study in the Greek population. Nutr Metab Cardiovasc Dis. 2016;26: 201–206. 10.1016/j.numecd.2015.10.003 26803594

[54] Chen N, Bezzina R, Hinch E, Lewandowski PA, Cameron-Smith D, Mathai ML, et al Green tea, black tea, and epigallocatechin modify body composition, improve glucose tolerance, and differentially alter metabolic gene expression in rats fed a high-fat diet. Nutr Res. 2009;29: 784–793. 10.1016/j.nutres.2009.10.003 19932867

[55] Vlavcheski F, Naimi M, Murphy B, Hudlicky T, Tsiani E. Rosmarinic Acid, a Rosemary Extract Polyphenol, Increases Skeletal Muscle Cell Glucose Uptake and Activates AMPK. Molecules. 2017;22: 1669. 10.3390/molecules22101669 PMC6151814 28991159

[56] Sayem A, Arya A, Karimian H, Krishnasamy N, Ashok Hasamnis A, Hossain C. Action of Phytochemicals on Insulin Signaling Pathways Accelerating Glucose Transporter (GLUT4) Protein Translocation. Molecules. 2018;23: 258. 10.3390/molecules23020258 PMC6017132 29382104

[57] Saxton RA, Sabatini DM. mTOR Signaling in Growth, Metabolism, and Disease. Cell. 2017;168: 960–976. 10.1016/j.cell.2017.02.004 28283069 PMC5394987

[58] Treins C, Giorgetti-Peraldi S, Murdaca J, Semenza GL, Van Obberghen E. Insulin Stimulates Hypoxia-inducible Factor 1 through a Phosphatidylinositol 3-Kinase/Target of Rapamycin-dependent Signaling Pathway. J Biol Chem. 2002;277: 27975–27981. 10.1074/jbc.M204152200 12032158

[59] Sang N, Stiehl DP, Bohensky J, Leshchinsky I, Srinivas V, Caro J. MAPK Signaling Up-regulates the Activity of Hypoxia-inducible Factors by Its Effects on p300. J Biol Chem. 2003;278: 14013–14019. 10.1074/jbc.M209702200 12588875 PMC4518846

[60] Bodine SC, Stitt TN, Gonzalez M, Kline WO, Stover GL, Bauerlein R, et al Akt/mTOR pathway is a crucial regulator of skeletal muscle hypertrophy and can prevent muscle atrophy in vivo. Nat Cell Biol. 2001;3: 1014–1019. 10.1038/ncb1101-1014 11715023

[61] Duriez C, Falette N, Audoynaud C, Moyret-Lalle C, Bensaad K, Courtois S, et al The human BTG2/TIS21/PC3 gene: genomic structure, transcriptional regulation and evaluation as a candidate tumor suppressor gene. Gene. 2002;282: 207–214. 11814693 10.1016/s0378-1119(01)00825-3

[62] Mo XY, Lan J, Jiao QZ, Xiong YZ, Zuo B, Li FE, et al Molecular characterization, expression pattern and association analysis of the porcine BTG2 gene. Mol Biol Rep. 2011;38: 4389–4396. 10.1007/s11033-010-0566-4 21116848

[63] Kashani A, Holman BWB, Nichols PD, Malau-Aduli AEO. Effect of dietary supplementation with Spirulina on the expressions of AANAT, ADRB3, BTG2 and FASN genes in the subcutaneous adipose and Longissimus dorsi muscle tissues of purebred and crossbred Australian sheep. J Anim Sci Technol. 2015;57: 8 10.1186/s40781-015-0047-3 26290728 PMC4540301

[64] Jiang H, Gautam SC, Jiang F, Pu P, Chopp M. Dietary Polyphenols as Preventive and Therapeutic Agents in: Glioblastoma. Springer New York; 2010 pp. 325–339.

[65] Juang H-H, Chang P-L, Tsui K-H. 148 B-cell translocation gene 2: A tumor suppressor gene is upregulated by resveratrol in bladder carcinoma cells. Eur Urol Suppl. 2013;12: e148.

[66] Walsh TG, Metharom P, Berndt MC. The functional role of platelets in the regulation of angiogenesis. Platelets. 2015;26: 199–211. 10.3109/09537104.2014.909022 24832135

[67] Lu J, Zhang K, Chen S, Wen W. Grape seed extract inhibits VEGF expression via reducing HIF-1a protein expression. Carcinogenesis. 2009;30: 636–644. 10.1093/carcin/bgp009 19131542 PMC2664452

[68] Daleprane JB, Schmid T, Dehne N, Rudnicki M, Menrad H, Geis T, et al Suppression of Hypoxia-Inducible Factor-1 Contributes to the Antiangiogenic Activity of Red Propolis Polyphenols in Human Endothelial Cells. J Nutr. 2012;142: 441–447. 10.3945/jn.111.150706 22279137

[69] Lamy S, Akla N, Ouanouki A, Lord-Dufour S, Béliveau R. Diet-derived polyphenols inhibit angiogenesis by modulating the interleukin-6/STAT3 pathway. Exp Cell Res. 2012;318: 1586–1596. 10.1016/j.yexcr.2012.04.004 22522122

[70] Gutierrez-Salmean G, Ciaraldi TP, Nogueira L, Barboza J, Taub PR, Hogan M, et al Effects of (−)-epicatechin on molecular modulators of skeletal muscle growth and differentiation. J Nutr Biochem. 2014;25: 91–94. 10.1016/j.jnutbio.2013.09.007 24314870 PMC3857584

[71] Kaminski J, Lançon A, Tili E, Aires V, Demarquoy J, Lizard G, et al Dietary resveratrol modulates metabolic functions in skeletal muscle cells. J Food Drug An. 2012;20: 398–401.

[72] Dugdale HF, Hughes DC, Allan R, Deane CS, Coxon CR, Morton JP, et al The role of resveratrol on skeletal muscle cell differentiation and myotube hypertrophy during glucose restriction. Mol Cell Biochem. 2018;444: 109–123. 10.1007/s11010-017-3236-1 29189984 PMC6002440

[73] Peoples GE, McLennan PL. Long-chain n-3 DHA reduces the extent of skeletal muscle fatigue in the rat in vivo hindlimb model. Br J Nutr. 2014;111: 996–1003. 10.1017/S0007114513003449 24229620

[74] Jeromson S, Gallagher IJ, Galloway SDR, Hamilton DL. Omega-3 fatty acids and skeletal muscle health. Mar Drugs. 2015;13: 6977–7004. 10.3390/md13116977 26610527 PMC4663562

[75] Smith GI, Julliand S, Reeds DN, Sinacore DR, Klein S, Mittendorfer B. Fish oil-derived n-3 PUFA therapy increases muscle mass and function in healthy older adults. Am J Clin Nutr. 2015;102: 115–122. 10.3945/ajcn.114.105833 25994567 PMC4480667

[76] Huguet A, Medja F, Nicole A, Vignaud A, Guiraud-Dogan C, Ferry A, et al Molecular, physiological, and motor performance defects in DMSXL mice carrying >1,000 CTG repeats from the human DM1 locus. PLoS Genet. 2012;8: e1003043 10.1371/journal.pgen.1003043 23209425 PMC3510028

[77] Collins JF, Bai L, Ghishan FK. The SLC20 family of proteins: dual functions as sodium-phosphate cotransporters and viral receptors. Pflügers Arch Eur J Physiol. 2004;447: 647–652. 12759754 10.1007/s00424-003-1088-x

[78] Mayer MP, Bukau B. Hsp70 chaperones: cellular functions and molecular mechanism. Cell Mol Life Sci. 2005;62: 670–84. 10.1007/s00018-004-4464-6 15770419 PMC2773841

[79] Robichon C, Varret M, Le Liepvre X, Lasnier F, Hajduch E, Ferré P, et al DnaJA4 is a SREBP-regulated chaperone involved in the cholesterol biosynthesis pathway. Biochim Biophys Acta—Mol Cell Biol Lipids. 2006;1761: 1107–1113. 10.1016/j.bbalip.2006.07.007 16950652

[80] Daugaard M, Rohde M, Jäättelä M. The heat shock protein 70 family: Highly homologous proteins with overlapping and distinct functions. FEBS Lett. 2007;581: 3702–3710. 10.1016/j.febslet.2007.05.039 17544402

[81] Mika D, Richter W, Westenbroek RE, Catterall WA, Conti M. PDE4B mediates local feedback regulation of β₁-adrenergic cAMP signaling in a sarcolemmal compartment of cardiac myocytes. J Cell Sci. 2014;127: 1033–1042. 10.1242/jcs.140251 24413164 PMC3937773

[82] Taipale M, Tucker G, Peng J, Krykbaeva I, Lin Z-Y, Larsen B, et al A quantitative chaperone interaction network reveals the architecture of cellular protein homeostasis pathways. Cell. 2014;158: 434–448. 10.1016/j.cell.2014.05.039 25036637 PMC4104544

[83] Kawabata S, Ishita Y, Ishikawa Y, Sakurai H. Immediate-early response 5 (IER5) interacts with protein phosphatase 2A and regulates the phosphorylation of ribosomal protein S6 kinase and heat shock factor 1. FEBS Lett. 2015;589: 3679–3685. 10.1016/j.febslet.2015.10.013 26496226

[84] Arndt V, Dick N, Tawo R, Dreiseidler M, Wenzel D, Hesse M, et al Report Chaperone-Assisted Selective Autophagy Is Essential for Muscle Maintenance. Curr Biol. 2010;20: 143–148. 10.1016/j.cub.2009.11.022 20060297

[85] Ramayo-Caldas Y, Ballester M, Sánchez JP, González-Rodríguez O, Revilla M, Reyer H, et al Integrative approach using liver and duodenum RNA-Seq data identifies candidate genes and pathways associated with feed efficiency in pigs. Sci Rep. 2018;8: 1–11. 10.1038/s41598-017-17765-5 29323241 PMC5764994

[86] Han X, Shen T, Lou H. Dietary polyphenols and their biological significance. International Journal of Molecular Sciences. 2007;8: 950–988.

[87] Surai F P. Antioxidant Systems in Poultry Biology: Superoxide Dismutase. J Anim Res Nutr. 2016;1: 8.

[88] Jakobek L. Interactions of polyphenols with carbohydrates, lipids and proteins. Food Chem. 2015;175: 556–567. 10.1016/j.foodchem.2014.12.013 25577120

