# Supplementary material for: Functional analysis finds differences on the muscle transcriptome of pigs fed an n-3 PUFA-enriched diet with or without antioxidant supplementations
Source: PLoS One. 2019 Feb 20;14(2):e0212449. doi: 10.1371/journal.pone.0212449 (PMC6382273; doi:10.1371/journal.pone.0212449)
Supplement: S1 Table — (DOCX) [file pone.0212449.s001.docx]

**S1 Table.** List of the genes tested by RT-qPCR in this study.

| **Gene name** | **Acronym** | **Primers** | **TM^(1)^(°C)** |
| --- | --- | --- | --- |
| *Peroxisome Proliferator-Activated Receptor Gamma Coactivator 1α* | *PPARGC1A* | F TAAAGATGCCGCCTCTGACT | 66 |
|  |  | R CTGCTTCGTCGTCAAAAACA |  |
| *Transforming Growth Factor Beta-induced* | *TGFBI* | F ACCGTCATGGCACGATTTTT | 66 |
|  |  | R ACAGGGTCACACGGAAGAGT |  |
| *Lipoprotein Lipase* | *LPL* | F CTGCTCCTAGTGGCTCTGAG | 67 |
|  |  | R CTCCTGAAATTCTGTCGGCG |  |
| *Thrombospondin 1* | *THBS1* | F GCGTTGGTGATGAGACAGAA | 64 |
|  |  | R CAAAGCAAGGATTGGACAGG |  |
| *Retinoid X Receptor Alpha* | *RXRA* | F CGAGCCCAAGACCGAGACG | 66 |
|  |  | R ACCAGGGTGAAGAGCTGCT |  |
| *Beta-2-Microglobulin* | *B2M* | F CCTTCTGGTCCACACTGAGT | 66 |
|  |  | R TCCCACTTAACTATCTTGGGCT |  |
| *Hypoxanthine Phosphoribosyltransferase 1* | *HPRT1* | F CCCAGCGTCGTGATTAGTGA | 66 |
|  |  | R CCTTTTCCAAATCCTCGGCA |  |

^(1)^TM = annealing temperature.
